# Golgi-Bypass Is a Major Unconventional Route for Translocation to the Plasma Membrane of Non-Apical Membrane Cargoes in *Aspergillus nidulans*

**DOI:** 10.3389/fcell.2022.852028

**Published:** 2022-04-07

**Authors:** Sofia Dimou, Mariangela Dionysopoulou, Georgia Maria Sagia, George Diallinas

**Affiliations:** ^1^ Department of Biology, National and Kapodistrian University of Athens, Panepistimioupolis, Athens, Greece; ^2^ Institute of Molecular Biology and Biotechnology, Foundation for Research and Technology, Heraklion, Greece

**Keywords:** traffic, secretion, polarity, fungi, COPII, endoplasmic reticulum, Pma1, pH sensing

## Abstract

Nutrient transporters have been shown to translocate to the plasma membrane (PM) of the filamentous fungus *Aspergillus nidulans via* an unconventional trafficking route that bypasses the Golgi. This finding strongly suggests the existence of distinct COPII vesicle subpopulations, one following Golgi-dependent conventional secretion and the other directed towards the PM. Here, we address whether Golgi-bypass concerns cargoes other than nutrient transporters and whether Golgi-bypass is related to cargo structure, size, abundance, physiological function, or polar vs. non-polar distribution in the PM. To address these questions, we followed the dynamic subcellular localization of two selected membrane cargoes differing in several of the aforementioned aspects. These are the proton-pump ATPase PmaA and the PalI pH signaling component. Our results show that neosynthesized PmaA and PalI are translocated to the PM *via* Golgi-bypass, similar to nutrient transporters. In addition, we showed that the COPII-dependent exit of PmaA from the ER requires the alternative COPII coat subunit LstA, rather than Sec24, whereas PalI requires the ER cargo adaptor Erv14. These findings strengthen the evidence of distinct cargo-specific COPII subpopulations and extend the concept of Golgi-independent biogenesis to essential transmembrane proteins, other than nutrient transporters. Overall, our findings point to the idea that Golgi-bypass might not constitute a fungal-specific peculiarity, but rather a novel major and cargo-specific sorting route in eukaryotic cells that has been largely ignored.

## Introduction

In eukaryotes, newly made plasma membrane (PM) proteins are thought to be directly sorted from ribosomes to the membrane of the endoplasmic reticulum (ER) *via* a co-translational translocation process (Voorhees and Hegde, 2016). After ER translocation, PM proteins are sorted into nascent ER-exit sites (ERes) and enter into COPII secretory vesicles, which fuse to the *early-* or *cis*-Golgi and then reach the *late-* or *trans*-Golgi network (TGN) *via* Golgi maturation (Zanetti et al., 2012; D’Arcangelo et al., 2013; Feyder et al., 2015; Gomez-Navarro and Miller, 2016; Casler et al., 2019). From the TGN, membrane proteins destined for the PM are thought to be secreted *via* AP-1/clathrin-coated vesicles, either directly or indirectly through the endosomal compartment, *via* a mechanism controlled by multiple Rab GTPases, a process also requiring microtubule and actin polymerization (Robinson, 2015; Zeng et al., 2017). However, this conventional secretory route of PM proteins has been characterized by studies on a limited number of specific transmembrane or extracellularly secreted cargoes. Noticeably, very little is known on how transporters and receptors, the two most abundant types of PM proteins, are translocated to the PM. In fact, several findings challenge the long-standing mechanism of export from the ER in small COPII-coated vesicles, reporting alternative mechanisms of unconventional protein secretion (UPS) that either do not use COPII components, bypass the Golgi, or exit the TGN in carriers other than the standard AP-1/clathrin-coated vesicles (Rabouille, 2017; Gee et al., 2018). In addition, recently proposed models also challenge the role of COPII in coating ER-budding vesicles, proposing that COPII helps to select secretory cargo, but does not coat the membrane carriers leaving the ER. Notably, these models propose that budding from the ER takes place *via tubular* structures or *tunnels* fusing with distal compartments (Phuyal and Farhan, 2021; Raote and Malhotra, 2021; Shomron et al., 2021; Weigel et al., 2021).

The discovery of alternative membrane cargo trafficking mechanisms opens new questions. The most obvious of these is how cargo specificity for these pathways is determined. Does size, oligomerization state, abundance, or targeting to specific PM micro-domains of cargoes drive distinct ER-exit mechanisms and trafficking to the PM? A cargo-centric view of ER-exit, which constitutes the first step in cargo trafficking, also opens the issue of whether distinct cargoes interact with specific ER-associated *trans*-effectors (e.g., *adaptors, chaperones,* and *v-SNARES*) to form structurally and functionally distinct COPII subpopulations (vesicles, tubules, or tunnels) and how this might be achieved. Experimental evidence supporting the existence of distinct subpopulations of COPII vesicles has been reported, but studies on the issue are limited. Several reports concern observations that GPI-anchored proteins are selectively accumulated in ERes distinct from those of other cargo proteins (Muñiz et al., 2001; Morsomme et al., 2003; Castillon et al., 2009; Bonnon et al., 2010). Other reports have shown that Sec24 isoforms (paralogs) are selective towards distinct cargoes (Roberg et al., 1999; Miller et al., 2002) or are non-uniformly distributed to ERes in *S. cerevisiae* (Iwasaki et al., 2015). In addition, in the moss *Physcomitrella patens*, COPII Sec23 isoforms have been shown to form specific ER-exit sites with differential effects on polarized growth (Chang et al., 2021). A hypothesis that cargo identity may define the formation of distinct COPII complexes and vesicle populations during plant development and in response to stress has also been proposed (Tanaka et al., 2013; Chung et al., 2016). A particular case concerns COPII vesicles involved in the ER-exit of bulky cargoes, which are too big to fit into ‘canonical’ COPII carriers. In the case of collagen, for example, co-packaging of specific cargo receptors, such as TANGO1 or cTAGE5 and also the COPII initiating factor Sec12 (normally excluded from small COPII vesicles), has been shown to drive the formation of large COPII-coated vesicles (Saito et al., 2009; Yuan et al., 2018; Raote and Malhotra, 2021).

In recent years, we have developed a controllable genetic system for studying membrane cargo trafficking in *Aspergillus nidulans*, a filamentous fungus that emerges as a powerful organism for studying cell biology *in vivo* (Scazzocchio, 2009; Diallinas, 2016; Steinberg et al., 2017; Dimou and Diallinas, 2020; Etxebeste and Espeso, 2020; Pinar and Peñalva, 2021). Through this system we established, in line with results from other fungal groups, that membrane proteins necessary for growth (e.g., cell wall or PM biosynthesis enzymes) follow the conventional secretory route to be *polarly* positioned at the growing tip of elongating cells (*germlings* and *hyphae*). These studies also showed that continuous local endocytic recycling of *apical* cargoes is essential to conserve their localization and function at the tip. Surprisingly, however, our most recent study, using as model membrane cargoes nutrient transporters, which are evenly distributed in the hyphal PM, challenged the concept of Golgi-dependent secretion as the sole major route for membrane cargo subcellular trafficking (Dimou et al., 2020). More specifically, we have obtained experimental evidence that the trafficking of neosynthesized transporters, after COPII-depended exit from the ER, occurs *via* Golgi-bypass and is independent of conventional post-Golgi secretion [e.g., AP-1 clathrin adaptor, Rab11 GTPase, or microtubule polymerization; (Robinson, 2015; Zeng et al., 2017; Pinar and Peñalva, 2021)]. These findings strongly support the existence of distinct, cargo-specific, ER-exit mechanisms and predict the existence of alternative COPII subpopulations.

Here, we examine whether the trafficking of neosynthesized membrane cargoes, other than nutrient transporters, also bypasses the Golgi. The rationale of cargoes selected to be studied is given in the *Results* section. Our study identifies two novel cargoes bypassing the Golgi, namely, the proton-pump ATPase PmaA and the PalI pH sensing component, reinforcing the concept that Golgi-bypass reflects a major mechanism of membrane trafficking in eukaryotic cells. Furthermore, we provide evidence for the existence of distinct cargo-specific COPII carriers based on the observation that the ER-exit of PmaA and PalI necessitates different cargo receptors (Sec24 vs. LstA), while PalI also requires the ER adaptor Erv14.

## Results

### Rationale of Cargoes Selected to be Studied and Experimental Design

**FIGURE 1:**
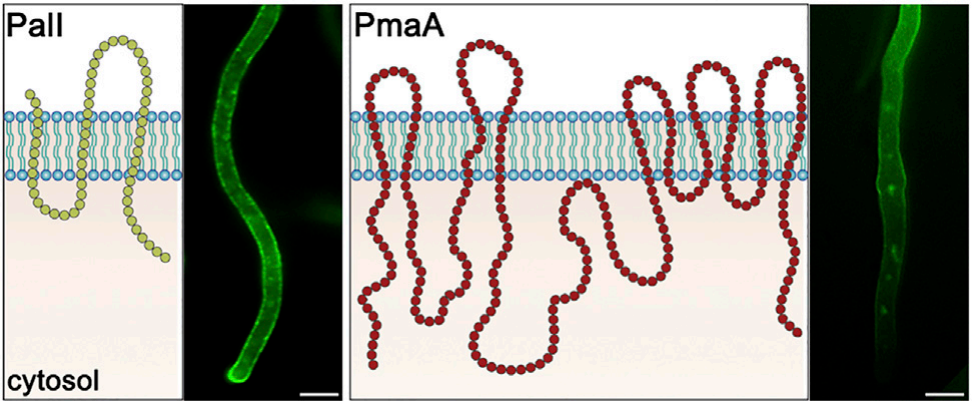
PM topology and subcellular localization of PalI and PmaI. PalI is a 3-TMS protein, with an N-terminal signal peptide and a C-basic terminus facing the cytosol (Calcagno-Pizarelli et al., 2007). PalI-GFP appears evenly distributed to the PM with a prominent apical polarization. PmaA is a 10-TMS protein, with both termini located in the cytosol (Ferreira et al., 2001). PmaA-GFP localizes homogenously to the PM of subapical hyphal regions and is almost absent from the growing apical tip. Scale bars: 5 μm.

Our primary question addressed in this study was whether Golgi-bypass concerns the sorting of membrane cargoes other than nutrient transporters and whether this mechanism is related to cargo structure, size, abundance, physiological function, or polar (apical) *vs.* non-polar (non-apical) distribution in the PM. To answer these questions, we selected two major *A. nidulans* PM cargoes differing in several of the aforementioned aspects. These are the main H^+^ pump ATPase PmaA^Pma1^, which is essential for the PM electrogenic potential needed for the functioning of transporters, and enzymes, regulation of pH, and cell homeostasis (Reoyo et al., 1998; Ambesi et al., 2000), and PalI^Rim9^, a component of the tripartite complex involved in signaling a response to an ambient pH value (Peñalva et al., 2014). These cargoes differ in size, number of transmembrane segments, oligomerization status, and essentiality for the cell. Previous studies have shown that PalI is not only non-polarly distributed along the entire PM but also forms cortical puncta of undefined nature (see also Figure 1). PmaA localization has not been studied in *A. nidulans*, but indirect evidence from studies of PmaA homologs in *Neurospora crassa* and *Saccharomyces cerevisiae* also point to a non-polar distribution along the PM (Fajardo-Somera et al., 2013; Henderson et al., 2014), as will also be confirmed herein (Figure 1).

We followed the dynamic subcellular localization of functional fluorescent-tagged versions of these cargoes, expressed from controllable promoters, in wild-type and mutant genetic backgrounds conditionally blocked in steps of the conventional secretory pathway. More specifically, we used mutant backgrounds where the transcriptional expression of key proteins involved in COPII formation (Sec24 or Sec13), early (SedV^Sed5^, GeaA^Gea1^) or late Golgi (HypB^Sec7^) functioning, post-Golgi vesicle formation (RabE^Rab11^, AP-1^σ^, ClaH^Clh1^), and sorting or recycling endosome functioning (RabA/B^Rab5^) can be tightly repressed (in superscript names of well-studied true orthologs in *S. cerevisiae*). Transcriptional repression of these factors was achieved using alleles where native promoters of the endogenous genes were replaced by the thiamine-repressible *thiA*_*p*_ promoter, as described in detail by Dimou et al. (2020) and in brief in *Materials and methods*. In all cases, the trafficking of neosynthesized cargoes was examined after the establishment of repression and depletion of factors essential for conventional Golgi-dependent trafficking, as shown by Dimou et al. (2020). In addition, we studied the trafficking of neosynthesized PmaA and PalI in the presence of drugs leading to depolymerization of tubulin (benomyl) or actin (latrunculin), also as described by Dimou et al. (2020). Finally, we performed co-localization studies of these two cargoes with key fluorescent molecular markers of the conventional secretory pathway, such as SedV, PH^OSBP^, and RabE, as established by Dimou et al. (2020). Results obtained were compared with those from a nutrient transporter (e.g., UapA) and a standard non-polar cargo, SynA (synaptobrevin secretory v-SNARE). The results obtained are described and discussed in the next sections.

### PmaA Translocation to the PM Bypasses the Conventional Golgi-Dependent Route

**FIGURE 2:**
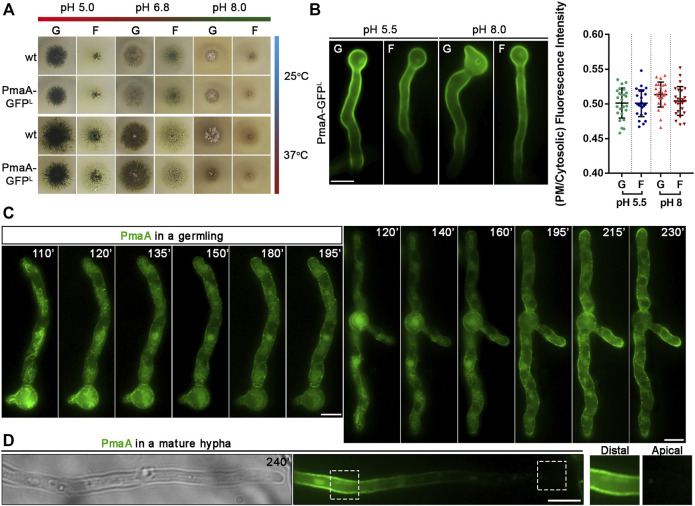
Growth phenotypes of strains expressing PmaA-GFP and subcellular localization at different development stages or pH. **(A)** Comparative growth test analysis of a strain expressing the GFP-tagged version of PmaA with an isogenic wild-type control strain on selected C sources [G: 1% (w/v) glucose; F: 0.1% (w/v) fructose] at 25°C or 37°C, at pH 5, 6.8, or 8. It is noted that the growth rate and morphology of the strain expressing the in-locus GFP-tagged version of PmaA are identical to those of the control strain, in all conditions tested. **(B)** Epifluorescence microscopy of a strain expressing the in-locus GFP-tagged version of PmaA at acidic (pH 5.5) or alkaline pH (pH 8) with glucose [G: 1% (w/v)] or fructose [F: 0.1% (w/v)] as the sole C source. It is noted that PmaA-GFP fluorescence is identical in all conditions. Results shown are confirmed by quantification (right panel) of PmaA-GFP PM/cytosolic intensity ratios for all conditions tested (for details, see *Materials and Methods*). Mean intensity ratios of PmaA-GFP are 0.501 ± 0.022 in glucose pH 5.5, 0.501 ± 0.019 in fructose pH 5.5, 0.513 ± 0.018 in glucose pH 8, and 0.504 ± 0.021 in fructose pH 8. For the statistical analysis, Tukey’s multiple comparison test was performed (one-way ANOVA). No statistically significant differences were found between different conditions. Biological/technical replicates for each condition: 2/25. **(C)** Epifluorescence microscopy of newly synthesized PmaA-GFP in two germlings after derepression of transcription (110–230 min). PmaA appears firstly in a membranous mesh and various cytosolic puncta (110–140 min) and steadily labels cortical puncta and finally the entire PM. **(D)** In mature hyphae, PmaA is practically no longer found in the PM of the apical region. Scale bars: 5 μm.

PmaA-GFP was expressed initially *via* its native promoter in a strain where the endogenous gene had been replaced *via* targeted homologous recombination with a GFP-tagged version of PmaA. The wild-type-like phenotype of this strain strongly suggests that the PmaA-GFP is functional in all growth media and pH tested (Figure 2A). Under epifluorescence microscopy, PmaA-GFP showed the expected PM-associated localization at all developmental stages tested (germinating conidiospores, germlings, and hyphae; Figure 2B; Supplementary Figure S1). As the main aspect of our study is the trafficking of newly synthesized membrane cargoes, we asked whether different physiological conditions might regulate the abundance of PmaA to the PM at a level that could be visibly monitored by epifluorescence microscopy. In *S. cerevisiae*, Pma1 is highly regulated by the presence of glucose, both transcriptionally and post-translationally, and by the decrease to the intracellular pH (Serrano, 1983; Cyert and Philpott, 2013). In our case, cellular expression and high steady-state levels of PmaA proved to be similar in minimal media differing in the carbon source or/and pH values (Figure 2B). The absence of glucose induction of PmaA expression was mentioned previously not only for *A. nidulans* (Abdallah et al., 2000) but also for its ortholog in *Penicillium simplicissimum* (Burgstaller et al., 1997), unlike what has been reported in *S. cerevisiae.* Overall, these results confirmed that GFP tagging has not affected PmaA cellular expression and PM localization and function and that PmaA expression is constitutive in *A. nidulans*.

In order to follow the localization of *de novo* made PmaA and given the constitutive expression of native PmaA, we replaced its endogenous promoter with the regulatable *alcA*_*p*_ promoter. *alcA*_*p*_ has been used previously for regulating the expression of other cargoes (Martzoukou et al., 2015; Dimou et al., 2020). In brief, transcription *via alcA*_*p*_ is tightly repressed in the presence of glucose, but derepressed upon a shift of cells to fructose media. Levels of proteins expressed upon *alcA*_*p*_ derepression are considered moderate. The strain expressing *alcA*_*p*_-PmaA-GFP was used to examine the dynamic localization of *de novo*-made PmaA-GFP in single growing cells at an early stage of development (e.g., germlings) and mature hyphae. Figure 2C shows that, after derepression of transcription, PmaA first localizes in a membranous mesh and few static cytosolic puncta (best seen at 110–140 min) and progressively labels more abundant cortical puncta (>140–230 min), to eventually label the entire PM in a rather homogenous manner (230 min). This picture, obtained in all cells examined (>100), resembles the one observed with nutrient transporters, rather than those obtained with apical cargoes involved in polar growth (for a comparison with the dynamics of localization of transporters and apical markers, see Dimou et al., 2020). Thus, PmaA seems to label an ER-like membrane mesh, rather than small Golgi-like foci. We also notice that, while in germlings PmaA labeled the growing tip (see 195–230 min), in more mature hyphae it was absent from the growing apical region (Figure 2D). The almost absent fluorescence from the tip area comes in agreement with observations of PMA-1-GFP localization in *N. crassa* (Fajardo-Somera et al., 2013). This localization is similar to nutrient transporters, and as reasoned by Dimou et al. (2020), strongly suggests that in mature hyphae neosynthesized PmaA and transporters are directly localized in the PM *via* lateral translocation from internal membranes, rather than being sorted to the apical tip and then diffusing laterally to the posterior PM.

**FIGURE 3:**
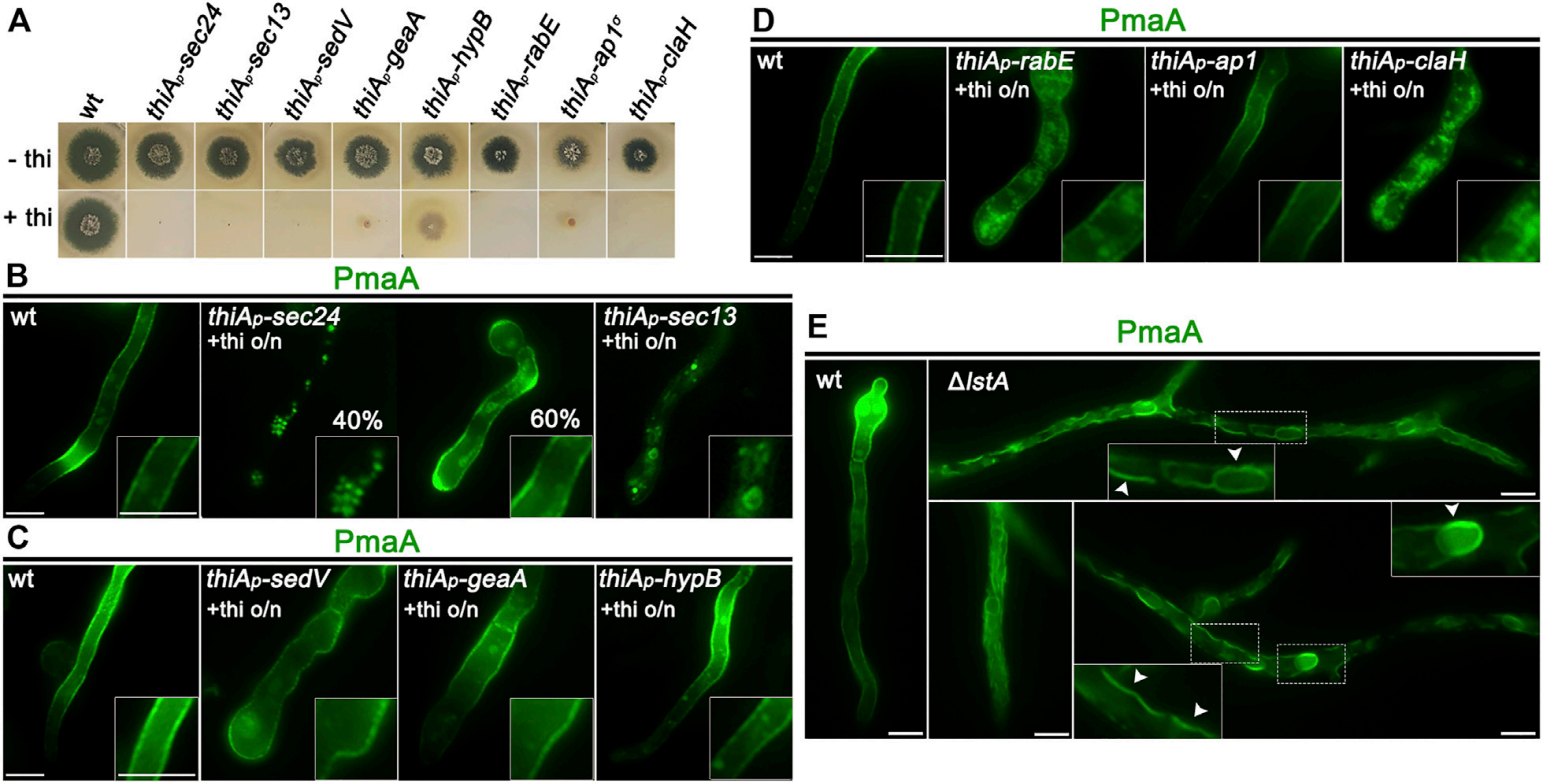
Subcellular localization of neosynthesized PmaA in trafficking mutant backgrounds. **(A)** Growth tests showing that, under repressing conditions (presence of thiamine in the growth medium), strains selected to follow the *de novo* expression of PmaA-GFP do not form proper colonies, confirming that all genes expressed under the *thiA*_*p*_ promoter are tightly repressed. Under derepressing conditions (absence of thiamine from the growth medium), all strains grow almost as the isogenic wild-type control strain (upper row). **(B)** Epifluorescence microscopic analysis of *de novo*-made PmaA-GFP in strains where *sec24* and *sec13* transcription is *ab initio* repressed by thiamine. o/n (overnight) means addition of thiamine from the onset of germination. The total lack of PM-associated signal of PmaA under conditions of Sec13 repression is noted, while Sec24 repression affects the ER-exit of PmaA in only ∼40% of the cell population (n = 92). **(C)** Epifluorescence microscopic analysis of *de novo*-made PmaA-GFP in strains where *sedV*, *geaA*, or *hypB* transcription is *ab initio* repressed by thiamine. It is noted that repression of these key Golgi proteins does not affect at all the proper localization of PmaA-GFP to the PM. **(D)** Epifluorescence microscopic analysis of *de novo*-made PmaA-GFP in strains where *rabE*, *ap-1* ^*σ*^, or *claH* transcription is *ab initio* repressed by thiamine. Notice that when *rabE* or *ap-1* ^*σ*^ is repressed, PmaA-GFP still reaches the PM, but in the latter, there is a clear accumulation of membranous cytoplasmic structures. Repression of *claH* totally abolishes the labeling of the PM and leads to PmaA retention in the cytosolic membrane or aggregates. **(E)** Epifluorescence microscopic analysis of *de novo*-made PmaA-GFP in strains where *lstA* is knocked out. In this case, PmaA fails to reach the PM and remains instead in the ER. The accumulation of PmaA inside the cortical ER and perinuclear ER rings is noted, as highlighted by white arrows. Scale bars: 5 μm.

The strain expressing PmaA-GFP from *alcA*_*p*_ was genetically crossed with strains carrying *thiA*_*p*_ *-*repressible alleles of key proteins involved in Golgi functioning and conventional cargo secretion. Appropriate isogenic progeny carrying *alcA*_*p*_-PmaA-GFP and repressible trafficking alleles were selected and were used to further study the sorting mechanism of PmaA. The corresponding strains do not form colonies under repressing conditions (presence of thiamine in the growth medium), except for *thiA*_*p*_-hypB which forms a slow-growing colony, although HypB is not detected in western blot analysis under repressing conditions (Figure 3A; Dimou et al., 2020). Figures 3B–E show representative results obtained with several cells in each experiment (n > 100). In all cases, we followed the final localization of PmaA-GFP upon 300 min of *de novo* expression, initiated after depletion of key proteins involved in conventional trafficking [i.e., overnight 14–16 h of growth in the presence of thiamine; Dimou et al. (2020)]. Thus, PmaA localization was performed for 5 h in cells where the conventional trafficking pathway was severally repressed. Notice that, in our conditions, upon repression of the conventional secretory pathway, *A. nidulans* cells stop growing and their apical regions swell, but remain alive for at least 10–12 h (Dimou et al., 2020). Collectively, our results show that PmaA is localized to the PM in all repressible trafficking mutants used, except *thiA*_*p*_-Sec13 and *thiA*_*p*_-ClaH (Figures 3B–D). Noticeably, however, repression of RabE (i.e., in *thiA*_*p*_-RabE) led to significant accumulation of cytoplasmic structures resembling membrane aggregates, suggesting the possible indirect involvement of RabE in the efficiency of the localization of PmaA to the PM (Figure 3D). Overall, the picture obtained with PmaA expressed in repressible trafficking mutant backgrounds was very similar to that of nutrient transporters, except in the case of *thiA*_*p*_-Sec24, where PmaA translocation to the PM was defective in only 40% of cells (see Figure 3B).

The partial independence of PmaA localization from Sec24 expression, not seen with the trafficking of nutrient transporters, which are fully dependent on Sec24 for PM localization (Dimou et al., 2020), suggested that PmaA might also be recognized by an alternative cargo receptor in specific COPII complexes. In *S. cerevisiae*, the ortholog of PmaA, Pma1, is indeed recognized by the Sec24 paralog Lst1, although both receptors co-operate in its ER-exit (Shimoni et al., 2000; Geva et al., 2017). Thus, we considered whether there is a true ortholog of Lst1 in *A. nidulans* and whether this operates in the PmaA ER-exit and further traffic to the PM. An *in silico* search and phylogenetic analysis showed that the product of AN3080 (https://fungidb.org) is a putative Lst1 ortholog and was, thus, named LstA (Supplementary Figure S2A, upper left panel). We knocked out (KO) the *lstA* gene by standard reverse genetics and showed that the mutant was viable, albeit showing a severe growth defect, more prominent at acidic conditions (Supplementary Figure S2A, upper right panel). *alcA*_*p*_-PmaA-GFP was introduced by genetic crossing in the background of Δ*lstA* and PmaA subcellular localization was followed, as described before. Figure 3E shows that the lack of LstA led to retention of PmaA-GFP in perinuclear and cortical ER membranes. The reduced flux of PmaA out of the ER is also compatible with the sensitivity of Δ*lstA* to low pH (Supplementary Figure S2A, upper right panel), as also reported in *S. cerevisiae* (Roberg et al., 1999)*.* Thus, COPII vesicles carrying PmaA include mostly LstA, rather than Sec24, similar to *S. cerevisiae*, which in turn suggests that they are distinct in composition from COPII vesicles carrying nutrient transporters or polar markers studied up to date in *A. nidulans.*

**FIGURE 4:**
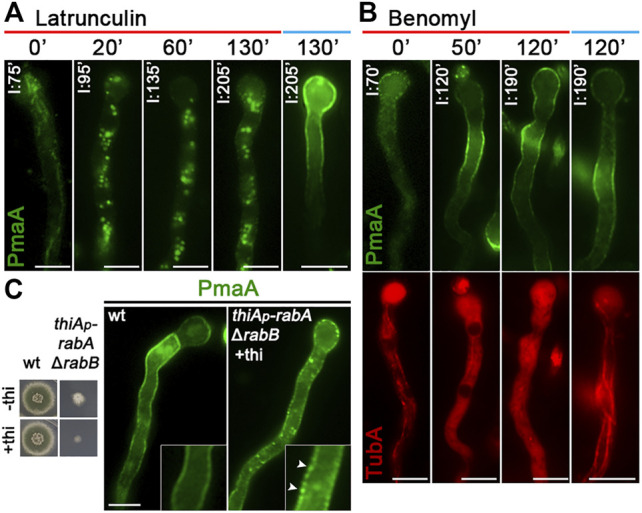
Role of actin, tubulin, or Rab5 endosome function in PmaA subcellular localization. **(A)** Time course of treatment with the actin polymerization drug latrunculin B for 0–130 min of a strain expressing neosynthesized PmaA-GFP under conditions of derepression compared to an untreated strain included as control. In all cases, latrunculin B was added at 75 min of PmaA derepression so that the total time of PmaA-GFP expression was 75–230 min in the different samples. It is noted that PmaA fails to reach the PM when latrunculin is present in the medium, in contrast to the control untreated strain. **(B)** Time course of treatment of strains expressing neosynthesized PmaA-GFP and mCherry-TubA with benomyl for 0–120 min. The anti-microtubule drug was added at 70 min of PmaA derepression so that the total time of PmaA-GFP expression was 70–190 min. Depolymerization of microtubules upon treatment with benomyl was evident by the diffused cytoplasmic signal of mCherry-TubA. It is noted that, in this case, PmaA normally reaches the PM. **(C)** Localization of newly synthesized PmaA-GFP in the absence of both RabA and RabB. *rabA* transcription is *ab initio* repressed (o/n) *via* thiamine, while *rabB* is genetically deleted. The double knockout/knockdown strain fails to form a proper colony in the presence of thiamine, as seen in the growth test on the left. It is noted that PmaA localization to the PM is not affected by the absence of proper endosomal functioning. Scale bars: 5 μm.

The localization of *de novo*-made PmaA to the PM was abolished when actin polymerization was blocked by latrunculin B but was not affected when microtubule polymerization was blocked by benomyl (Figures 4A,B). PmaA localization to the PM was also examined when Rab5-dependent endosomes were repressed or knocked out (i.e., in *thiA*_*p*_ *-rabA/*Δ*rabB* double mutant). Figure 4C shows that when the functioning Rab5 endosomes were blocked, the great majority of PmaA still reached the PM normally. Although the appearance of a few cytoplasmic PmaA-GFP in *thiA*_*p*_ *-rabA/*Δ*rabB* foci might suggest a minor role of Rab5-like endosomes in PmaA exocytosis or recycling, this is in sharp contrast with the absolute PM delocalization effect that the depletion of Rab5-containing endosomes has on some polar secreted cargoes (Hernández-González et al., 2018b).

**FIGURE 5:**
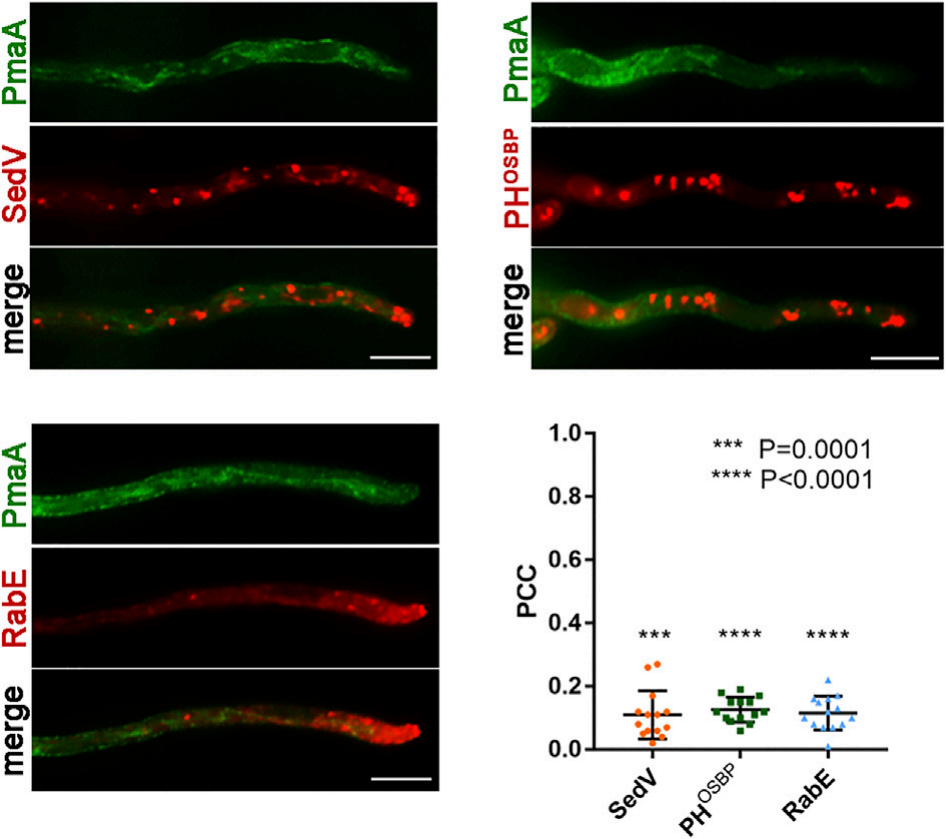
Co-localization of PmaA with Golgi markers. Co-localization analysis and relevant quantification of strains co-expressing *de novo*-made PmaA-GFP with mCherry-SedV, mRFP-PH^OSBP^, and mRFP-RabE. Quantification by calculating Pearson’s correlation coefficient (PCC) shows a clear non-co-localization of PmaA with all the Golgi markers tested, as confirmed by the one-sample *t*-test (PCC = 0.11 ± 0.08 with ***p = 0.0001 for SedV, PCC = 0.13 ± 0.04 with ****p < 0.0001 for PH^OSBP^, and PCC = 0.12 ± 0.05 with ****p < 0.0001 for RabE). Biological/technical replicates: 3/14 for each strain.

To obtain further evidence supporting the Golgi-bypass of *de novo*-made PmaA translocation to the PM, we performed a series of key co-localization studies with established red fluorescent protein markers of the early (mCherry-SedV) and late (mRFP-PH^OSBP^) Golgi, as well as of post-Golgi vesicles (mRFP-RabE). The necessary isogenic strains were constructed by standard crossing, as described in *Materials and methods*. Notice that, in these experiments, we follow the *ab initio* dynamic co-localization of PmaA with the other protein markers, rather than recording its terminal localization to the PM. Figure 5 shows that PmaA-GFP did not co-localize with any of the three molecular markers used, unlike what had been reported for conventional Golgi-dependent cargoes (i.e., SynA; see Dimou et al., 2020). Overall, our results strongly supported that PmaA, similar to nutrient transporters, does not follow the conventional post-Golgi trafficking route to localize in the PM.

### PalI Translocation to the PM Bypasses the Conventional Golgi-Dependent Route

**FIGURE 6:**
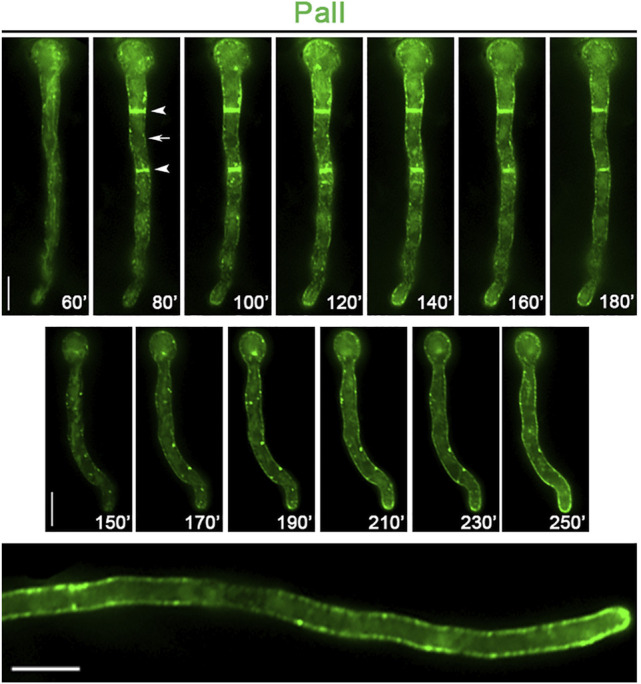
Subcellular localization of neosynthesized PalI. *In vivo* epifluorescence microscopy of *de novo*-made PalI-GFP observed in two single germlings at various times (60–250 min, upper and middle panels) after transcriptional derepression. PalI initially labels a membranous network (best seen at 60 min in the upper panel) and later ER rings and septa (see arrows in the upper panel). Notice also the frequent appearance of cytosolic or cortical immotile foci (better detected at 150–230 min in the middle panel). In a more mature hypha, PalI (360 min) is homogenously localized at the plasma membrane, with still some prominent cortical foci (lower panel). Scale bars: 5 μm.

As in the case of PmaA, in order to regulate the expression of PalI and follow the translocation of neosynthesized protein to the PM, we replaced the constitutive and a very weak endogenous promoter with *alcA*_*p*_ (i.e., *alcA*_*p*_-PalI-GFP). Previous studies by the Peñalva group have also used a similar construct to study the role of PalI pH sensing (Calcagno-Pizarelli et al., 2007). We first followed the *de novo* appearance of PalI-GFP upon derepression in single cells. Figure 6 shows representative results (n = 100), which revealed that PalI initially labels a clear membranous network, coincident with a few cortical or cytoplasmic puncta (60–80 min), and progressively accumulates more and more in cortical foci all along hyphal PM, with a rather homogenous distribution (100–180 min). In some samples, PalI also appears early in septa (80 min), indicating a very fast PM localization. In more grown cells (250 min), PalI labels the entire PM with still some distinct cortical foci, as also shown in the work of Calcagno-Pizarelli et al. (2007).

**FIGURE 7:**
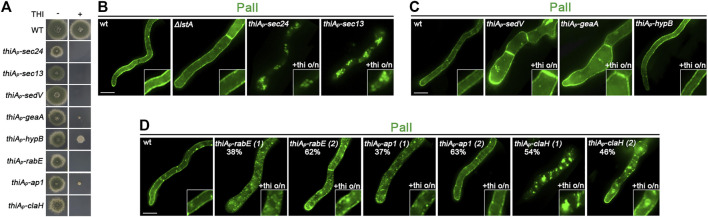
Subcellular localization of neosynthesized PalI in trafficking mutant backgrounds. **(A)** Growth tests showing that, in the presence of thiamine in the growth medium, strains selected to study the *do novo* localization of PalI-GFP do not form proper colonies, confirming that all genes expressed under the *thiA*_*p*_ promoter are tightly repressed. In the absence of thiamine from the growth medium (derepressed conditions), the corresponding strains grow nearly as an isogenic wild-type control. **(B)** Epifluorescence analysis of *de novo*-made PalI-GFP when the transcription of *sec24* or *sec13* is repressed or *lstA* is genetically deleted. Transcription of *sec24* and *sec13* was *ab initio* blocked by the addition of thiamine in the media. It is noted that PalI is properly localized at the PM in the Δ*lstA* background, whereas it is dramatically abolished when *sec24* and *sec13* are repressed. **(C)** Epifluorescence analysis of *de novo*-made PalI-GFP when *sedV*, *geaA*, or *hypB* transcription is repressed by *ab initio* addition of thiamine. PalI-GFP translocates to the PM in all cases. **(D)** Epifluorescence analysis of *de novo*-made PalI-GFP when *rabE, ap-1* ^*σ*^, or *claH* transcription is repressed by *ab initio* addition thiamine. PalI translocation to the PM was detected when *rabE* or *ap-1* ^*σ*^ was repressed in the majority of germlings or hyphae (n = 100). In a minority of hyphae, PalI-GFP labeled static cytoplasmic structures, possibly membranous aggregates. Repression of *claH* transcription significantly blocked PalI-GFP translocation to the PM in the majority of cells. Scale bars: 5 μm.

**FIGURE 8:**
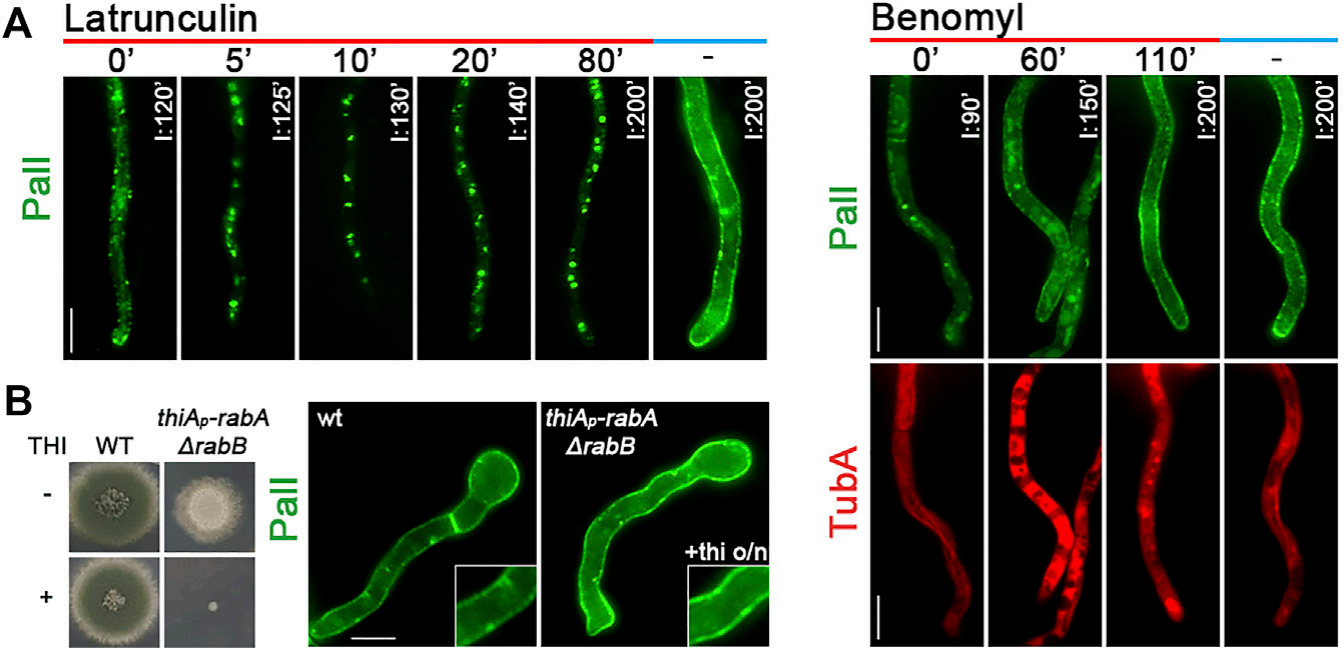
Effect of actin or tubulin depolymerization in PalI subcellular localization. **(A) *Left panel***: time course treatment with the actin depolymerization drug latrunculin B for 0, 5, 10, 20, or 80 min of a strain expressing neosynthesized PalI-GFP under conditions of derepression compared to an untreated strain included as control (200 min). Latrunculin B was added at 120 min of derepression so that the total time of PalI-GFP expression was 120, 125, 130, 140, and 200 min in the different samples. The abolishment of sorting of PalI to the PM after 5 min of latrunculin B addition is noted in the growth medium. ***Right panel***: time course treatment of strains co-expressing neosynthesized PalI-GFP and mCherry-TubA with the anti-microtubule drug benomyl, for 0, 60, or 110 min. In all cases, benomyl was added at 90 of PalI derepression so that the total time of PalI-GFP expression was 90, 150, or 200 min. Benomyl abolished the thread-like appearance of microtubules in all samples added, evident by the diffuse cytoplasmic signal of mCherry-TubA. It is noted that PalI normally reaches the PM (best detected at 200 min of derepression). **(B)** When Rab5-dependent endosomes were knocked down/knocked out (i.e., in *thiA*_*p*_-*rabA*/Δ*rabB*), fungal growth was arrested (as depicted in the growth test shown at the left panel); however, PalI-GFP was properly localized at the PM (right panel). Scale bars: 5 μm.

The strain expressing *alcA*_*p*_-PalI-GFP was genetically crossed with strains carrying selected *thiA*_*p*_ *-*repressible alleles of key proteins involved in Golgi functioning and conventional cargo secretion, as described for PmaA. Appropriate isogenic progeny carrying *alcA*_*p*_-PalI-GFP and repressible alleles were used to study the trafficking route of PalI. Most of the selected strains did not form proper colonies in the presence of thiamine in the growth medium, confirming the efficient repression of genes involved in Golgi-dependent trafficking in all cases (Figure 7A). Figures 7B–D show representative results obtained from several cells in each experiment (n > 100). In all cases, we followed the final localization of PalI-GFP upon 300 min of *de novo* expression, initiated after the full repression of trafficking protein expression (14–16 h of growth in the presence of thiamine). PalI localization to the PM was found to be absolutely dependent on Sec24, Sec13, or clathrin heavy chain (ClaH), but fully independent of SedV, GeaA, and HypB, and partially affected by RabE or AP-1. PalI translocation to the PM was also independent of LstA, in line with its full dependence on Sec24. Through the use of cytoskeleton polymerization drugs, PalI localization proved to be actin dependent, but microtubule and Rab5-like endosome independent, as shown previously for PmaA and nutrient transporters (Dimou et al., 2020) (Figures 8A,B).

**FIGURE 9:**
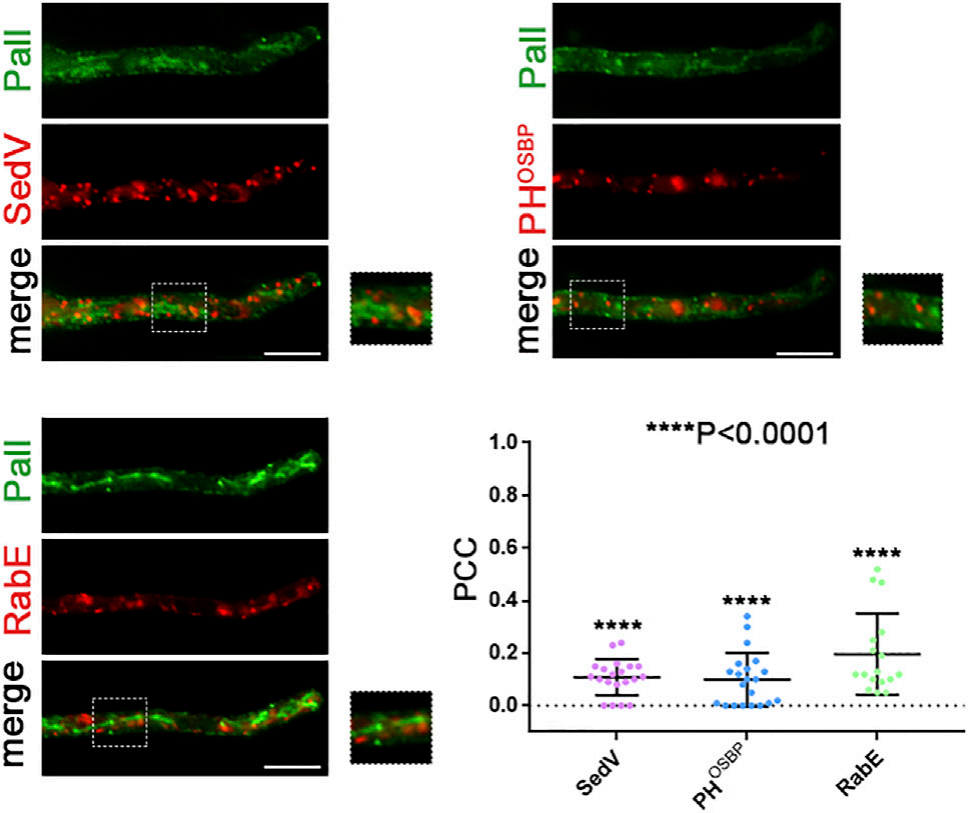
Co-localization study of PalI with early and late Golgi markers. Localization of *de novo*-made PalI-GFP in three different strains co-expressing mCherry-SedV, mRFP-PH^OSBP^, or mRFP-RabE in single hyphae. Quantification (lower right panel) by calculating Pearson’s correlation coefficient shows no significant co-localization of PalI with the Golgi markers tested, as confirmed by the one-sample *t*-test (PCC = 0.11 ± 0.07, 0.1 ± 0.10, or 0.2 ± 0,15, with ****p < 0.0001 for mCherry-sedV, PH^OSBP^, or RabE respectively). Biological/technical replicates: 2/20, 2/21, and 2/17 for each strain respectively.

Further evidence supporting the Golgi-bypass of *de novo*-made PalI translocation to the PM was obtained through co-localization studies with key fluorescent markers of the early (mCherry-SedV) and late (mRFP-PH^OSBP^) Golgi, or of conventional post-Golgi vesicles (mRFP-RabE). The necessary isogenic strains were constructed as described in *Materials and methods*. Figure 9 shows that PalI-GFP does not co-localize significantly with the Golgi or post-Golgi markers used.

Collectively, our findings confirm that neosynthesized PalI traffics to the PM *via* a Golgi-independent route that shares similar features with that employed by neosynthesized nutrient transporters and PmaA. Thus, the sole evident differences related to Golgi-bypass cargoes concerned the COPII cargo adaptor involved (Sec24 *vs.* LstA) and the level of dependence on the post-Golgi effectors RabE or AP-1.

### The Erv14 Cargo Adaptor Is Essential for PalI ER-Exit

**FIGURE 10:**
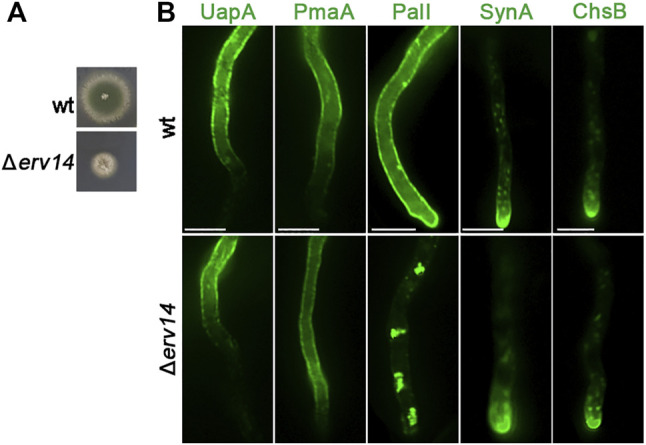
Growth phenotype of Δerv14 and subcellular localization of UapA, PmaA, PalI, SynA, and ChsB in Δerv14. **(A)** Colony growth of Δ*erv14* compared to an isogenic wild-type control strain (wt). **(B)** Epifluorescence microscopic analysis of non-polar and polar cargoes in conditions of Erv14 depletion (lower panel). It is noted that the lack of Erv14 does not affect the localization of UapA, PmaA, SynA, and ChsB, but leads to the retention of PalI in cytosolic aggregates. Scale bars: 5 μm.

Erv14 is a COPII accessory protein involved in specific cargo packaging and vesicle formation in yeast (Powers and Barlowe, 2002). This cargo receptor belongs to the well-conserved Erv14/cornichon protein family (Erv14 in yeast, cornichon in *Drosophila*, and CNIH in mammals) which mediates the ER export of many transmembrane proteins, such as plasma membrane permeases, anti-porters, and multi-drug transporters (Herzig et al., 2012; Pagant et al., 2015; Rosas-Santiago et al., 2017). We examined whether the homologs of Erv14 have a crucial role in the ER-exit of Golgi bypassers and/or conventional apical cargoes. We, thus, identified *via in silico* searches the single homolog of Erv14 of *A. nidulans* as the product of the gene-annotated AN5195 (Supplementary Figure S2B). We constructed the KO *erv14* mutant by standard reverse genetics (Δ*erv14*). Figure 10A depicts its growth phenotype, showing a significantly reduced rate of growth and altered colony morphology. Δ*erv14* could germinate to germlings and hyphae in liquid cultures, in line with the viability of the analogous null mutant in yeast. Δ*erv14* was crossed with strains expressing not only GFP-tagged PmaA or PalI but also the UapA transporter, as another Golgi-bypasser, and SynA or ChsB, which are standard Golgi-dependent cargoes. Appropriate progeny from these crosses was used for studying the effect Erv14 on the trafficking of these cargoes. Erv14 expression was crucial only for PalI trafficking, as in its absence PalI showed extremely reduced translocation to the PM, concomitant with the appearance of fluorescent cytosolic aggregates, most probably the result of ER-associated aberrant accumulation (Figure 10B). No other cargo tested showed dependence on Erv14 (Figure 10B). This is in line with reports in yeast or mammals showing that cargo receptor complexes involving Erv-like proteins are required for export from the ERes of only some specific cargoes (Herzig et al., 2012). Overall, our results showed that Erv14 acts as a crucial and specific cargo receptor of PalI during COPII formation, but is dispensable for all other cargoes tested.

## Discussion

A major question that has arisen from the recent discovery showing that *de novo* made transporters, after COPII-dependent exit from the ER, find their way to the PM without passing from the Golgi and without employing a conventional post-Golgi mechanism, is whether other membrane proteins can also use the same unconventional route to be targeted to the PM. The present study has given a definite answer to this question. By selecting two well-characterized major membrane cargoes, the H^+^ pump ATPase PmaA and the pH-sensing PalI component, we showed that both proteins bypass the Golgi and translocate to the PM without the need of microtubule polymerization or endosomal functioning.

Overall, our previous (Dimou et al., 2020) and present results show that several nutrient transporters, the major H^+^ pump ATPase PmaA and a component of a pH sensor, all use a similar Golgi-independent route to translocate from the ER to the PM. This route is clearly distinct from the well-established Golgi- and microtubule-dependent trafficking of cargoes needed for *A. nidulans* polar growth, including chitin synthase ChsB (Fukuda et al., 2009; Hernández-González et al., 2018a), the synaptobrevin-like secretory v-SNARE SynA (Taheri-Talesh et al., 2008; Pantazopoulou and Peñalva, 2011; Martzoukou et al., 2018), the lipid flippases DnfA and DnfB (Schultzhaus et al., 2015, 2017; Martzoukou et al., 2018), the glycosylphosphatidylinositol-anchored protein (GPI-AP) EglC (Peñalva et al., 2020), or the soluble extracellular inulinase InuA (Hernández-González et al., 2018b). The membrane cargoes SynA, ChsB, DnfA, and DnfB all show strict polar localization at the Spitzenkörper/SPK [a vesicle supply apical center; (Zhou et al., 2018)] and the apical plasma membrane, co-localize dynamically with Golgi markers, and their biogenesis is aberrant in conditional mutants of key Golgi proteins (e.g., SedV, HypA, HypB, Tlg2, or RabO). Polarization of these cargoes occurs by direct vesicular sorting (i.e., Rab5 endosome independent), coupled with endocytic recycling at a subapical endocytic region and subsequent trafficking to TGN. The periplasmically secreted EglC enzyme, which also requires functional key Golgi proteins for its trafficking (e.g., TRAPPII complex and RabE-dependent), is translocated in a polarized fashion but then rapidly redistributes towards apico-distal regions. The extracellularly secreted InuA also follows the conventional Golgi-dependent secretory pathway, its secretion being blocked in temperature-sensitive mutants of *sedV, rabO, hypA,* or *hypB*. The biogenesis of all aforementioned cargoes necessitates functional COPIIs and seems microtubule dependent. Thus, the overall picture emerging from previous studies and results presented herein and by Dimou et al. (2020) is that the mechanism of trafficking and the steady-state localization of polarly secreted cargoes and Golgi-bypassers are markedly different. This difference seems to be related to physiological functions, as polar cargoes are related to biosynthesis or modification of cell wall and plasma membrane and are thus restricted in apical tips (Steinberg et al., 2017; Martzoukou et al., 2018), whereas Golgi bypassers serve cell nutrition and pH homeostasis in all compartments of hyphae and are thus sorted all over the PM. In other words, conventional, Golgi-dependent, trafficking seems to serve polar growth, while Golgi-bypass concerns nutrient supply and cell homeostasis.

Interestingly, the steady state, Golgi-dependent, anti-polar localization of EglC in hyphae somehow resembles that of nutrient transporters and PmaA, which are Golgi bypassers. It has been suggested that EglC is delivered from apical regions to basolateral parts of hyphae *via* an unknown mechanism. This seems quite feasible as this cargo accumulates in the outer space of the PM and, thus, might freely diffuse in the periplasm. This mechanism of anti-polar diffusion of a soluble cargo is not compatible with Golgi bypassers for several reasons. First, all Golgi bypassers studied are large oligomerizing transmembrane proteins. For example, PmaA is a ∼100 kDa transmembrane protein forming 6-12mers in the PM (Zhao et al., 2021), UapA is a 123 kDa tight dimer which might oligomerize further (Martzoukou et al., 2015; Alguel et al., 2016), and PalI is part of the heterocomplex of ∼180 kDa (Calcagno-Pizarelli et al., 2007). It is strongly unlikely that such protein oligomers will diffuse rapidly long distances (>100 μm) within a lipid bilayer (Valdez-Taubas and Pelham, 2003). Second, transporters, PmaA and PalI appear in the PM in a non-continuous manner at several apical-distant cortical foci, rather than forming a gradient from the apex. In fact, transporters and PmaA are absent from apical regions of mature hyphae, dismissing the idea of lateral diffusion from the apex of growing cells. Finally, it is rather hard to consider that very long hyphal cells would translocate transporters, PmaA and PalI exclusively at their growing tips, while posterior distant parts of the cell are ‘striving’ to adapt to environmental fluctuations in nutrient availability and pH.

In an article from 2011, it has been speculated that, in *S. cerevisiae,* Pma1 is sorted to the Golgi before translocation to the PM (Huang and Chang, 2011). However, there is no formal evidence in this report for Golgi-dependent biogenesis of *de novo*-made Pma1. On the contrary, Huang and Chang (2011) have shown that Pma1 translocates properly to the PM in null *aps1*Δ mutants (i.e., absence of the AP-1 function), which are defective in the formation of post-Golgi vesicles, which also suggests the existence of a Golgi-independent sorting route for Pma1 in yeast. Notably, several reports stating that a cargo is sorted *via* the Golgi do not distinguish neosynthesized from recycling fractions, which might explain why Golgi-bypass has been overlooked in membrane trafficking studies. Notice also that yeast Pma1 and Rim9 (the yeast ortholog of PalI) are non-glycosylated proteins (Holcomb et al., 1988; Chang and Slayman, 1991; Obara et al., 2012). It has also been reported that, in *S. cerevisiae,* lipid rafts help to carry Pma1 through the Golgi to the plasma membrane (Bagnat et al., 2001). However, the entry of the proton ATPase into rafts and oligomerization seem to occur earlier, as raft-associated Pma1 oligomers can be isolated from COPII vesicles (Lee et al., 2002). PmaA, PalI, and nutrient transporters of *A. nidulans* are also not glycosylated. Absence of glycosylation is a strong indication that a protein does not pass from the Golgi. It is noticed, however, that the opposite is not true, as core N-glycosylation of membrane proteins takes place in the ER (Aebi, 2013).

Interestingly, an anti-polar localization (i.e., absence from growing apical regions) has also been observed for the orthologous H^+^ ATPase PMA-1 in *Neurospora crassa* (Fajardo-Somera et al., 2013). In this report, the authors state that PMA-1 traffics to the PM *via* the Golgi. However, their work does not distinguish neosynthesized from recycling PMA-1, the latter being sorted to the PM *via* the endosomal compartment and TGN. In contrast to the anti-polar localization of PmaA and nutrient transporters, PalI appears rather homogenously in apical and subapical regions (i.e., non-polar distribution). This suggests that PalI might be needed for proper apical growth. In fact, previous reports have provided evidence for the need of alkaline pH gradient for proper polarized growth of fungal hyphae (Robson et al., 1996). The authors of this report proposed that pH sensing is critical in regulating the local assembly of cytoskeletal components (e.g., actin) and specific vesicle tethering at the apex, required for hyphal extension. In Dimou et al. (2020), a possible mechanistic explanation has been proposed for the absence of transporters from the apical tips of hyphae, based on actin filament re-distribution during transition from germlings to hyphae. How PalI remains in the apical tip during growth, while PmaA and transporters are absent from tip areas, remains elusive.

The experimental evidence presented in our previous (Martzoukou et al., 2018; Dimou et al., 2020) and present reports show that multiple trafficking mechanisms co-exist to serve the differential physiological roles of cargoes and the mode of cell growth. As most animal and plant cells are polar, the identification of distinct Golgi-dependent and Golgi-independent trafficking mechanisms seemingly associated with polar and non-polar targeting might not be an *A. nidulans* or fungal particularity, but rather reflect two major cargo trafficking mechanisms present also in other eukaryotes. This idea is supported by reports showing that specific mammalian transporters might also bypass the Golgi under specific physiological or stress conditions. These include the insulin-regulated human glucose transporter GLUT4 (Camus et al., 2020), a mutant version of the CFTR transporter associated with cystic fibrosis (Gee et al., 2018), and a handful of specifically localized mammalian cargoes in neurons, such as glutamate receptor GluA1, neuroligin, or the potassium channel Kv2.1 (Arnold and Gallo, 2014; Bowen et al., 2017; Stampe Jensen et al., 2017). Recently, the ER chaperone BiP/HSPA5/GRP78, a major regulator of the unfolded protein response (UPR), has been found to accumulate in the PM, where it assumes novel functions associated with signal transduction and cancer metastasis, also *via* Golgi-bypass. In this case, PM translocation is mediated by Rab4/Rab11/Rab15 GTPases and necessitates the ER v-SNARE Bet1 and endosomal t-SNARE syntaxin 13, suggesting vesicular transfer from the ER to the PM *via* specialized endosomes (Van Krieken et al., 2021). Interestingly, a recent report provided evidence that the assembly and cellular secretion of coronaviruses and other budding viruses employs a direct connection of ERGIC with endosomes, bypassing the passage from Golgi stacks (Saraste and Prydz, 2021). Cargo Golgi-bypass has also been recently speculated in *Physcomitrella patens*, based on the observation that Sec23 isoforms form distinct and functionally specific COPII/ERes, with some of them affecting ER to Golgi trafficking and polarized growth (i.e., conventional secretion), while others proved unrelated to polarized growth, while affecting specific cargo secretion (Chang et al., 2021). In fact, the multiple isoforms of COPII components present in plants (Chung et al., 2016) and mammals (Jensen and Schekman, 2011) may point to functional diversity, rather than redundancy, related not only to the cargoes selected but also the trafficking route followed after ER-exit. Thus, Golgi-independent cargo trafficking is emerging as a major trafficking route of membrane proteins in eukaryotes, serving bulk and/or non-directional cargo sorting.

## Materials and Methods

### Media, Strains, Growth Conditions, and Transformation

Standard complete and minimal media for *A. nidulans* were used (FGSC, http://www.fgsc.net). Media and chemical reagents were obtained from Sigma-Aldrich (Life Science Chemilab SA, Hellas) or AppliChem (Bioline Scientific SA, Hellas). Glucose 1% (w/v) or fructose 0.1% (w/v) was used as the carbon source. NH_4_^+^(di-ammonium tartrate) and NaNO_3-_ were used as nitrogen sources at 10 mM. Thiamine hydrochloride was used at a final concentration of 10–20 μM as a repressor of the *thiA*_*p*_ promoter (Apostolaki et al., 2012) in microscopy or western blot analysis. *A. nidulans* transformation was performed by generating protoplasts from germinating conidiospores using TNO2A7 as a recipient strain that allows the selection of transformants *via* complementation of a pyrimidine autotrophy (Nayak et al., 2006). Integrations of gene fusions with fluorescent tags, promoter replacement fusions, or deletion cassettes were selected using the *A. fumigatus* markers orotidine-5-phosphate-decarboxylase (AF*pyrG*, Afu2g0836), GTP-cyclohydrolase II (AF*riboB*, Afu1g13300), or a pyridoxine biosynthesis gene (AF*pyroA*, Afu5g08090), resulting in the complementation of the relevant auxotrophies. Transformants were verified by PCR and Southern analyses. Combinations of mutations and fluorescent epitope-tagged strains were generated by standard genetic crossing and progeny analysis. The *E. coli* strains used were DΗ5a. *A. nidulans* strains used are listed in Supplementary Table S1.

### Nucleic Acid Manipulations and Plasmid Constructions

Genomic DNA extraction was performed as described in FGSC (http://www.fgsc.net). All DNA fragments used in the various constructs were amplified from a TNO2A7 strain. Plasmid preparation and DNA gel extraction were performed using the Nucleospin Plasmid and the Nucleospin Extract-II kits (Macherey-Nagel, Lab Supplies Scientific SA, Hellas), restriction enzymes were from Takara Bio (Lab Supplies Scientific SA, Hellas), DNA sequences were determined by Eurofins-Genomics (Vienna, Austria), conventional PCRs and high-fidelity amplifications were performed using KAPA Taq DNA and Kapa HiFi polymerases (Kapa Biosystems, Roche Diagnostics, Hellas), and gene cassettes were generated by sequential cloning of the relevant fragments in the pGEM-T plasmid, which served as a template to PCR-amplify the relevant linear cassettes.

### Conditions Used to Repress–Derepress Cargo Expression

For following the subcellular trafficking and localization of *de novo* made PmaA-GFP or PalI-GFP, we used the regulatable *alcA*_*p*_ promoter (Waring et al., 1989) combined with a repression–derepression setup analogous to the one described by Dimou et al. (202) or by Calcagno-Pizarelli et al. (2007), respectively. In brief, cargo expression was repressed by overnight growth (for 12–14 h, at 25°C) in the presence of glucose as the sole carbon source and derepressed by a change to fructose (Dimou et al., 2020) or ethanol media (Calcagno-Pizarelli et al., 2007), for the following 1–8 h of growth. For following the trafficking of other control cargoes (UapA, ChsB, or SynA), we used an analogous setup. In experiments aiming at repressing key trafficking proteins expressed from the *thiA*_*p*_ promoter, 10 mM thiamine was used throughout growth. For the microscopic analysis of trafficking markers tagged with fluorescent epitopes, we used their native promoter (RabE), *alcA*_*p*_ (TubA), or the strong constitutive promoter *gpdA*_*p*_ (SedV, PH^OSBP^), as described by Dimou et al. (2020). All relevant strains carrying mRFP/mCherry-tagged versions of the trafficking markers were the product of in-locus gene replacements.

### Protein Extraction and Western Blots

Total protein extraction was performed as previously described by Dimou et al. (2020), using dry mycelia from cultures grown in minimal media supplemented with NaNO_3-_ at 25°C. Total proteins (50 μg, estimated by Bradford assays) were separated in a 6% (w/v) polyacrylamide gel and were transferred on PVDF membranes (GE Healthcare Life Sciences, Amersham). Immunodetection was performed with an anti-GFP monoclonal antibody (11814460001, Roche Diagnostics), an anti-actin monoclonal (C4) antibody (SKU0869100-CF, MP Biomedicals, Europe), and an HRP-linked antibody (7076, Cell Signaling Technology Inc.). Blots were developed using the Lumi Sensor Chemiluminescent HRP Substrate kit (Genscript, United States) and SuperRX Fuji medical X-Ray films (Fuji FILM, Europe).

### Fluorescence Microscopy and Statistical Analysis

Conidiospores were incubated overnight in glass-bottom 35 mm l-dishes (ibidi, Lab Supplies Scientific SA, Hellas) in liquid minimal media, for 16–22 h at 25°C, under conditions of transcriptional repression of cargoes expressed from the *alcA*_*p*_ promoter [1% (w/v) glucose or 10 mM NH_4_^+^] and repression of the selected trafficking proteins expressed under the *thiA*_*p*_ promoter (10 mM thiamine). Transcriptional derepression of cargoes was performed through a shift in media containing fructose or ethanol as sole carbon source for PmaA or PalI respectively. Derepression periods ranged from 60 min to 12 h, according to experiments. Benomyl (Sigma-Aldrich) and latrunculin B (Sigma-Aldrich) were used at 2.5 and 100 μg/ml final concentrations, respectively. To determine the effect of extracellular pH in PmaA-GFP fluorescence, conidiospores were cultured in an acidic (pH 5.5 with 25 mM KH_2_PO_4_) or alkaline medium (pH 8 with 23.5 mM K_2_HPO_4_, 0.15 mM KH_2_PO_4_). The images were obtained using an inverted Zeiss Axio Observer Z1 equipped with an Axio Cam HR R3 camera. Contrast adjustment, area selection, and colour combining were made using the Zenlite 2012 software. Scale bars were added using the FigureJ plugin of the ImageJ software. The images were further processed and annotated in Adobe Photoshop CS4 Extended version 11.0.2. Technical replicates correspond to different hyphal cells observed within each sample, while biological replicates correspond to different samples (Martzoukou et al., 2017). For quantifying co-localization (Dunn et al., 2011), Pearson’s correlation coefficient (PCC) above thresholds, for a selected region of interest (ROI), was calculated using the ICY co-localization studio plugin (pixel-based method) (http://icy.bioimageanalysis.org/). A one-sample *t*-test was performed to see whether the mean PCC value was significantly greater than 0, using the GraphPad Prism software (Mcdonald and Dunn, 2013). Confidence interval was set to 95%. For quantifying the fluorescence of PmaA in Figure 2, two ROIs in the same region were drawn manually, using the area selection tool in ICY, one including both the PM and the cytoplasm and another identical one, excluding the PM (Dimou et al., 2020). PM/cytoplasmic mean fluorescence intensity ratios for each condition are shown in box scatter plots, using the GraphPad Prism software. To test the significance of differences in PM/cytoplasmic fluorescence of measurements, Tukey’s multiple comparison test was performed (one-way ANOVA).

## Data Availability

The original contributions presented in the study are included in the article/Supplementary Material, further inquiries can be directed to the corresponding author.
