# Supplementary material for: Golgi-Bypass Is a Major Unconventional Route for Translocation to the Plasma Membrane of Non-Apical Membrane Cargoes in Aspergillus nidulans
Source: Front Cell Dev Biol. 2022 Apr 7;10:852028. doi: 10.3389/fcell.2022.852028 (PMC9021693; doi:10.3389/fcell.2022.852028)
Supplement: Supplementary file 1 [file DataSheet1.PDF]

Golgi-bypass is a major unconventional route for translocation to the plasma membrane of non-apical membrane cargoes in *Aspergillus nidulans*

Sofia Dimou[1]*, Mariangela Dionysopoulou[1]*, Georgia Maria Sagia[1], and George Diallinas[1,2#]

[1]Department of Biology, National and Kapodistrian University of Athens, Panepistimioupolis, 15784 Athens, Greece.

[2]Institute of Molecular Biology and Biotechnology, Foundation for Research and Technology, 70013 Heraklion, Greece.

*Equal contribution

#Correspondence to: diallina@biol.uoa.gr, tel. +30(210)7274649

Key words: traffic; secretion; traffic; polarity; fungi; COPII

Running title: Golgi bypass of a PM cargoes

Keywords: fungi, COPII, Endoplasmic Reticulum, Pma1, pH sensing

Supplementary Material

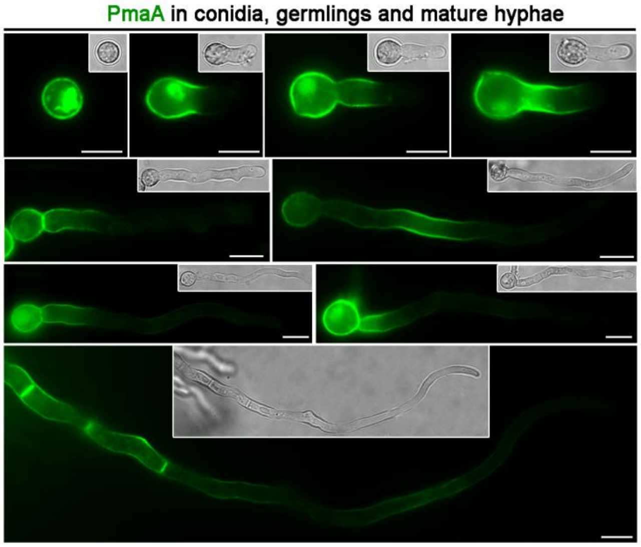

Figure S1. Localization of PmaA in germinating conidiospores, germlings and mature hyphae. In conidiospores, before germ tube emergence, PmaA-GFP labels homogenously the entire PM, but also internal cytoplasmic structures that might be the ER. In germinated conidiospores and germlings, PmaA-GFP is found all along the conidial PM and across to the germ tube, with the fluorescence becoming less apparent at the growing apex, which is the newest part of the cell. In mature hyphae, PmaA-GFP is localized at the PM at subapical hyphal regions and in septa, while it is almost absent from the PM of the hyphal tip.

A

Lst1				
Gene ID	Organism	Score	E-value	Protein Length
**AN3080**	***Aspergillus nidulans FGSC A4***	**400**	**5,00E-123**	**1031**
AN3720	*Aspergillus nidulans FGSC A4*	214	2,00E-58	910
AN4572	*Aspergillus nidulans FGSC A4*	33,1	2,60E-01	312
AN11874	*Aspergillus nidulans FGSC A4*	30,8	1,90E+00	1683
AN7432	*Aspergillus nidulans FGSC A4*	28,9	5,20E+00	219

Proteins	% Identity	% Similarity
Lst1 vs AN3080	27,3	43,3

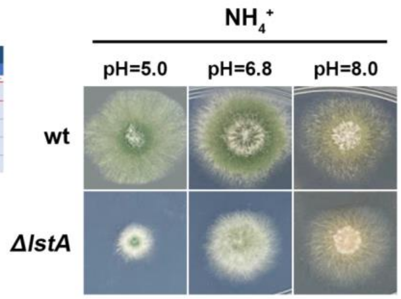

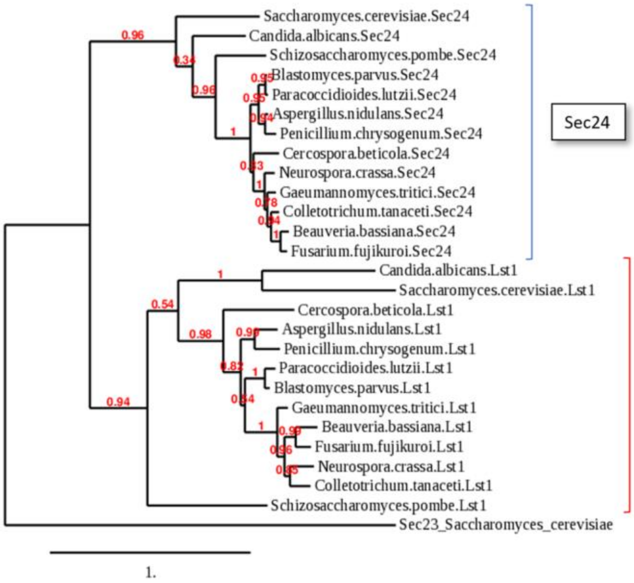

B

Erv14				
Gene ID	Organism	Score	E-value	Protein Length
**AN5195**	***Aspergillus nidulans FGSC A4***	**179**	**4,00E-58**	**138**
AN6493	*Aspergillus nidulans FGSC A4*	28,5	7,50E-01	765
AN8014	*Aspergillus nidulans FGSC A4*	27,3	1,50E+00	858
AN6329	*Aspergillus nidulans FGSC A4*	26,6	2,70E+00	3187
AN2520	*Aspergillus nidulans FGSC A4*	25	8,90E+00	377

Proteins	% Identity	% Similarity
Erv14 vs AN5195	60,0	75,7

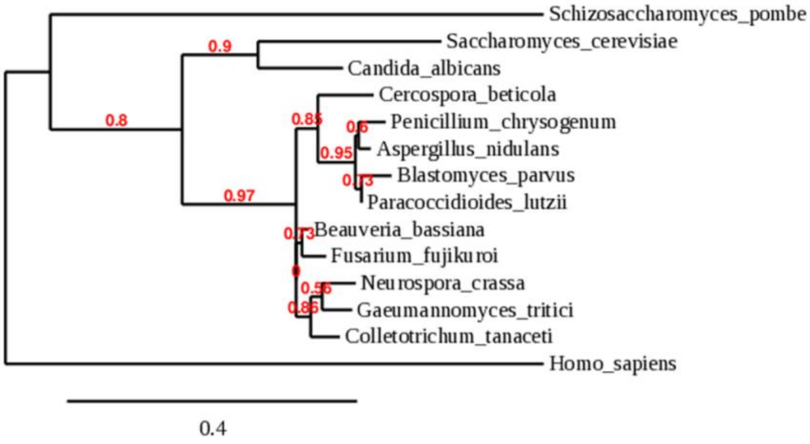

Figure S2. Evidence of homology between *A. nidulans* and *S. cerevisiae* proteins studied in this work. (A) Upper left panel: BlastP analysis for Lst1 with the NCBI blastP tool in FungiDB (https://fungidb.org) detects AN3080 as an *A. nidulans* homologue. Upper right panel: Growth test of a control strain (wt) and its isogenic Δ*lstA* null mutant which shows a pH-dependent growth defect. Lower panel: Phylogenetic analysis of Lst1 and Sec24 sequences from representative species of Ascomycota, showing that for each species there are distinct homologues of Sec24 and Lst1. **(B)** BlastP analysis of Erv14 from *S. cerevisiae* reveals homology with AN5195. Binary sequence identity/similarity of proteins analysed is shown that the bottom panel, as measured by EMBOSS needle (https://www.ebi.ac.uk/Tools/psa/emboss_needle/).

Table S1. Strains used in this study. *pabaA1*, *pyroA4*, *riboB2*, *argB2*, *pyrG89, pantoB100, biA1*, *nicA2* and *inoB2* are auxotrophic mutations for p-aminobenzoic acid, pyridoxine, riboflavin, arginine, uracil/uridine, D-pantothenic acid, biotin, nicotinic acid and inositol respectively. *yA2* and *wA3* or *wA4* are mutations resulting in yellow and white conidiospore colors respectively. *nkuA* is a gene essential for non-homologous end joining of DNA in double-strand break repair. Deletion of *nkuA* (Δ*nkuA*) greatly reduces the frequency of non-homologous integration of transforming DNA fragments, leading to dramatically improved gene targeting. All strains carry the veA1 mutation affecting sporulation. Δ7 is a strain carrying total deletions in seven genes encoding transporters involved nucleobase-related cellular import. All strains carrying more than one gene modified were constructed via standard genetic crossing of respective singly modified strains.

Strain	Genotype	Source
wt	*pabaA1*	wild-type reference strain
TNO2A7	*ΔnkuA::argB pyrG89 pyroA4 riboB2*	(Nayak et al., 2006)
Δ7	*ΔuapA ΔuapC::AFpyrG ΔazgA ΔfcyB::argB ΔfurD::AFriboB ΔfurA::AFriboB ΔcntA::AFriboB pantoB100 pabaA1*	(Krypotou et al., 2015)
Δ7 $alcA_p$-PalI-GFP	*ΔuapA ΔuapC::AFpyrG ΔazgA ΔfcyB::argB ΔfurD::AFriboB ΔfurAΔ:AFriboB ΔcntA::AFriboB pantoB100 pabaA1 pGEM-*$alcA_p$*-palI-GFP::panto*	This study
$alcA_p$-PalI-GFP	*pGEM-*$alcA_p$*-palI-GFP::panto ΔnkuA::argB riboB2 pyroA4*	This study
PmaA-GFP^{L}	*pmaA-(5xGA)GFP::AFpyrG ΔnkuA::argB pyrG89 riboB2 pyroA4*	This study
$alcA_p$-PmaA-GFP	*pGEM-*$alcA_p$*-pmaA-(5xGA)GFP pyrG89 argB2 pantoB100 pabaA1*	This study
$alcA_p$-PmaA-GFP $thiA_p$-Sec24	*pGEM-*$alcA_p$*-pmaA-(5xGA)GFP* $thiA_p$*-sec24::AFpyrG pabaA1*	This study
$alcA_p$-PmaA-GFP $thiA_p$-Sec13	*pGEM-*$alcA_p$*-pmaA-(5xGA)GFP* $thiA_p$*-sec13::AFpyrG pabaA1*	This study
$alcA_p$-PmaA-GFP $thiA_p$-SedV	*pGEM-*$alcA_p$*-pmaA-(5xGA)GFP* $thiA_p$*-sedV::AFpyrG pyrG89 pyroA4 pabaA1*	This study
$alcA_p$-PmaA-GFP $thiA_p$-GeaA	*pGEM-*$alcA_p$*-pmaA-(5xGA)GFP* $thiA_p$*-geaA::AFriboB pyrG89 pyroA4 pabaA1*	This study

$alcA_p$-PmaA-GFP $thiA_p$-HypB	*pGEM-*$alcA_p$*-pmaA-(5xGA)GFP* $thiA_p$*-hypB::AFpyrG pyrG89 pyroA4 pabaA1*	This study
$alcA_p$-PmaA-GFP $thiA_p$-RabE	*pGEM-*$alcA_p$*-pmaA-(5xGA)GFP* $thiA_p$*-rabE::AFpyrG pyrG89 pyroA4*	This study
$alcA_p$-PmaA-GFP $thiA_p$-Ap1	*pGEM-*$alcA_p$*-pmaA-(5xGA)GFP* $thiA_p^{FLAG}$*-*$ap1^{\sigma}$*::AFriboB pyrG89 pyroA4 pabaA1*	This study
$alcA_p$-PmaA-GFP $thiA_p$-ClaH	*pGEM-*$alcA_p$*-pmaA-(5xGA)GFP claH::*$thiA_p$*-claH::AFpyroA pyrG89 pabaA1*	This study
ΔLstA	*ΔlstA::AFriboB ΔnkuA::argB? pyrG89? pyroA4 riboB2?*	This study
$alcA_p$-PmaA-GFP ΔLstA	*pGEM-*$alcA_p$*-pmaA-(5xGA)GFP ΔlstA::AFriboB pyroA4*	This study
$alcA_p$-PmaA-GFP $alcA_p$-mCherry-TubA	*pGEM-*$alcA_p$*-pmaA-(5xGA)GFP* $alcA_p$*-mCH-tubA::pyroA pyroA4 pyrG89*	This study
$alcA_p$-PmaA-GFP $thiA_p$-RabA ΔRabB	*pGEM-*$alcA_p$*-pmaA-(5xGA)GFP pyrG89 argB2* $thiA_p$*-rabA::AfpyrG ΔrabB::AFpyroA pantoB100 pabaA1*	This study
$alcA_p$-PmaA-GFP mCherry-SedV	*pGEM-alcAp-pmaA-(5xGA)GFP pyroA4::[pyroAmut::*$gpdA^m_p$*::mCherry::sedV] pabaA1*	This study
$alcA_p$-PmaA-GFP mRFP-PH^{OSBP}	*pGEM-*$alcA_p$*-pmaA-(5xGA)GFP [pyroA-*$gpdA^m_p$*-mrfp-*PH^{OSBP}*]pyroA wA4 pabaA1*	This study
$alcA_p$-PmaA-GFP mRFP-RabE	*pGEM-*$alcA_p$*-pmaA-(5xGA)GFP mRFP-rabE::AFpyrG pyrG89 argB2 pantoB100 pabaA1*	This study
$alcA_p$-PalI-GFP ΔLstA	*ΔlstA::AFriboB pGEM-*$alcA_p$*-palI-GFP::panto*	This study
$alcA_p$-PalI-GFP $thiA_p$-Sec24	*ΔuapA? ΔuapC::AFpyrG? ΔazgA? ΔfcyB::argB? ΔfurD::AFriboB? ΔfurA::AFriboB? ΔcntA::AFriboB?* $thiA_p$*-sec24::AFpyrG pGEM-*$alcA_p$*-palI-GFP::panto pabaA1 pyroA4*	This study
$alcA_p$-PalI-GFP $thiA_p$-Sec13	*pGEM-*$alcA_p$*-palI-GFP::panto* $thiA_p$*-sec13::AFpyrG pyroA4*	This study
$alcA_p$-PalI-GFP $thiA_p$-SedV	$thiA_p$*-sedV::AFpyrG ΔnkuA::argB pGEM-*$alcA_p$*-palI-GFP::panto pyroA4 pantoB100*	This study
$alcA_p$-PalI-GFP $thiA_p$-GeaA	*pGEM-*$alcA_p$*-palI-GFP ΔnkuA::argB* $thiA_p$*-geaA::AFriboB pyroA4*	This study

alcA$_p$-PalI-GFP *thiA*$_p$-HypB	*thiA$_p$-hypB::AFpyrG pGEM-alcA$_p$-palI-GFP::panto*	This study
alcA$_p$-PalI-GFP *thiA*$_p$-RabE	*thiA$_p$-rabE::AFpyrG pGEM-alcA$_p$-palI-GFP::panto pabaA1 pyroA4*	This study
alcA$_p$-PalI-GFP *thiA*$_p$-Ap1	*pGEM-alcA$_p$-palI-GFP::panto ΔnkuA::argB thiA$_p$-ap1^β::Afribo pyroA4*	This study
alcA$_p$-PalI-GFP *thiA*$_p$-ClaH	*thiA$_p$-claH::AFpyroA pGEM-alcA$_p$-PalI-GFP::panto nicA2 pantoB100 pabaA1*	This study
alcA$_p$-PalI-GFP mCherry-SedV	*pGEM-alcA$_p$-palI-GFP::panto pyroA4::[pyroAmut::gpdA^m$_p$::mCherry::sedV] pantoB100 pabaA1*	This study
alcA$_p$-PalI-GFP mRFP-PH^{OSBP}	*pGEM-alcA$_p$-palI-GFP::panto PHosbp: [pyroA-gpdA^m$_p$-mRFP-PH^{OSBP}]pyroA4 inoB2*	This study
alcA$_p$-PalI-GFP mRFP-RabE	*pGEM-alcA$_p$-palI-GFP::panto mRFP-rabE::pyrg89 pabaA1 argB*	This study
alcA$_p$-PalI-GFP *alcA*$_p$-mCherry-TubA	*pGEM-alcA$_p$-palI-GFP ΔnkuA::argB alcA$_p$-mCH-tubA::pyroA*	This study
alcA$_p$-PalI-GFP ΔRabB *thiA*$_p$-RabA	*ΔrabB::AFpyroA pGEM-alcA$_p$-palI-GFP::panto ΔnkuA::bar thiA$_p$-rabA::AfpyrG pyrG89*	This study
ΔErv14	*Δerv14::AFriboB ΔnkuA:argB pyrG89 pyroA4 riboB2*	This study
UapA-GFP	*ΔuapA::uapA-GFP::AFriboB ΔuapC::AfpyrG ΔnkuA::argB pabaA1 pyroA4 riboB2*	(Evangelinos et al., 2016)
UapA-GFP ΔErv14	*Δerv14::AFpyrG ΔuapA::uapA-GFP^L ΔnkuA::argB pyrG89 pyroA4*	This study
alcA$_p$-PmaA-GFP ΔErv14	*pGEM-alcA$_p$-pmaA-(5xGA)GFP Δerv14::AFriboB pantoB100 pabaA1*	This study
alcA$_p$-PalI-GFP Δerv14	*pGEM-alcAp-palI-GFP ΔnkuA::argB Δerv14::AFriboB pyroA4*	This study
alcA$_p$-GFP-SynA	*alcA$_p$-GFP-synA::AFpyrG ΔnkuA::argB pyrG89 pyroA4 riboB2*	(Dimou et al., 2020)
alcA$_p$-GFP-SynA ΔErv14	*Δerv14::AFriboB alcA$_p$-GFP-synA::AFpyrG ΔnkuA:argB pyrG89 pyroA4*	This study

GFP-ChsB	*GFP-chsB::AFpyrG ΔnkuA::argB pyrG89 pyroA4 riboB2*	(Dimou et al., 2020)
GFP-ChsB ΔErv14	*Δerv14::AFriboB GFP-chsB::AFpyrG nkuAΔ::argB pyrG89 pyroA4 riboB2*	This study
